# Optimization of SARS-CoV-2 culture from clinical samples for clinical trial applications

**DOI:** 10.1128/msphere.00304-24

**Published:** 2024-10-16

**Authors:** Dominic Wooding, Kate Buist, Alessandra Romero-Ramirez, Helen Savage, Rachel Watkins, Daisy Bengey, Caitlin Greenland-Bews, Caitlin R. Thompson, Nadia Kontogianni, Richard Body, Gail Hayward, Rachel L. Byrne, Susan Gould, CONDOR Steering Group, A. Joy Allen, Christopher Myerscough, Barry Atkinson, Victoria Shaw, Bill Greenhalf, Emily Adams, Ana Cubas-Atienzar, Saye Khoo, Tom Fletcher, Thomas Edwards

**Affiliations:** 1 Centre for Drugs and Diagnostics, Liverpool School of Tropical Medicine and Hygiene, Liverpool, United Kingdom; 2 Department of Clinical Sciences, Liverpool School of Tropical Medicine and Hygiene, Liverpool, United Kingdom; 3 Manchester University NHS Foundation Trust, Research and Innovation, Manchester, United Kingdom; 4 Nuffield Department of Primary Care Health Sciences, University of Oxford, Oxford, United Kingdom; 5 Research and Evaluation, UK Health Security Agency, Porton Down, Salisbury, United Kingdom; 6 University of Liverpool, Liverpool, United Kingdom; 7 NIHR Royal Liverpool and Broadgreen CRF, Liverpool University Hospitals NHS Foundation Trust, Liverpool, United Kingdom; University of Michigan, Ann Arbor, Michigan, USA

**Keywords:** molecular diagnostics, clinical trials, COVID-19

## Abstract

Clinical trials of severe acute respiratory syndrome coronavirus 2 (SARS-CoV-2) therapeutics often include virological secondary endpoints to compare viral clearance and viral load reduction between treatment and placebo arms. This is typically achieved using quantitative reverse-transcriptase PCR (RT-qPCR), which cannot differentiate replicant competent virus from non-viable virus or free RNA, limiting its utility as an endpoint. Culture-based methods for SARS-CoV-2 exist; however, these are often insensitive and poorly standardized for use as clinical trial endpoints. We report optimization of a culture-based approach evaluating three cell lines, three detection methods, and key culture parameters. We show that Vero-angiotensin-converting enzyme 2-transmembrane serine protease 2 cells in combination with RT-qPCR of culture supernatants from the first passage provides the greatest overall detection of Delta viral replication (22 of 32, 68.8%), being able to identify viable virus in 83.3% (20 of 24) of clinical samples with initial Ct values of <30. Likewise, we demonstrate that RT-qPCR using culture supernatants from the first passage of Vero human signaling lymphocytic activation molecule cells provides the highest overall detection of Omicron viral replication (9 of 31, 29%), detecting live virus in 39.1% (9 of 23) of clinical samples with initial Ct values of <25. This assessment demonstrates that combining RT-qPCR with virological endpoint analysis has utility in clinical trials of therapeutics for SARS-CoV-2; however, techniques may require optimization based on dominant circulating strain.

**IMPORTANCE:**

RT-qPCR is commonly used for virological endpoints during clinical trials for antiviral therapy to determine the quantity and presence of virus in a sample. However, RT-qPCR identifies viral RNA and cannot determine if viable virus is present. Existing culture-based techniques for SARS-CoV-2 are insensitive and not sufficiently standardized to be employed as clinical study endpoints. The use of a culture system to monitor replicating viruses could mitigate the possibility of molecular techniques identifying viral RNA from inactive or lysed viral particles. The methodology optimized in this study for detecting infectious viruses may have application as a secondary virological endpoint in clinical trials of therapeutics for SARS-CoV-2 in addition to numerous research processes.

## INTRODUCTION

The design of COVID-19 therapeutic clinical trials and appropriate selection of viral endpoints is crucial in determining treatment efficacy (1). A variety of endpoints have been identified by systematic reviews of severe acute respiratory syndrome coronavirus 2 (SARS-CoV-2) trial endpoints, including death, recovery, need for intensive care, hospital discharge, oxygenation, critical illness assessment tools, and viral load assessment (1–3), and multiple secondary endpoints are often selected for analysis.

The use of viral load assays as an endpoint has also underpinned the early-phase evaluation of antiviral activity, such as remdesivir (4), molnupiravir (5), and nirmatrelvir (6). The gold standard method for detecting and quantifying SARS-CoV-2 in clinical samples is quantitative reverse-transcriptase PCR (RT-qPCR) detection of viral RNA (7). However, RT-qPCR cannot distinguish between infectious virus and non-infectious degraded RNA fragments that persist after neutralization by the immune system and, therefore, may overestimate the presence of infectious virus and underestimate efficacy of the assessed pharmaceutical. Previous studies have shown a correlation between viral load by RT-qPCR during SARS-CoV-2 infection and culture positivity, with culture positivity being used as an estimate of infectiousness (8).

Since these RNA-based detection assays do not discriminate between replication-competent virus and remnants of genetic material, an alternative approach is to use viral culture as a proxy for antiviral efficacy. While viral culture is less sensitive than RT-qPCR, it has the advantage of confirming viral infectivity and therefore transmission potential (9, 10). Culture-based methods for detecting SARS-CoV-2 are not well standardized, and numerous cell lines and culture conditions have been reported (11). SARS-CoV-2 cell line susceptibility is influenced by many factors including cell tropism, receptor expression levels, virus replication kinetics, and the epidemiological and clinical features of the virus (12). Globally, the Vero E6 African green monkey kidney cell line is commonly used as a readily available cell line that is permissive for infection by many viruses. Vero E6 cells do not express all SARS-CoV-2 key surface molecules, and viral entry and fusion mainly occur via non-specific endocytic mechanisms (13). Alternative Vero cells have been modified to more closely resemble the human epithelia., e.g., Vero human signaling lymphocytic activation molecule (hSLAM) cells that express the hSLAM (14), and Vero E6-ACE2-TMPRSS2 (VAT) cells-Vero E6 expressing both human angiotensin-converting enzyme 2 (ACE2), the major receptor of SARS-CoV-2, and transmembrane serine protease 2 (TMPRSS2), which cleaves the viral S protein priming it for cellular infection (15). Both viral growth kinetics and changes to cell morphology [i.e., cytopathic effects (CPEs)] can vary between these different cell lines thereby impacting the outcome of tests used to assess culture positivity (16). For example, a 2020 study found that cell culture supernatants from Vero E6 cells expressing TMPRSS2 had more than 100 times more viral RNA copies than Vero E6 cells not expressing this protein (17).

There are various methods for assessing culture positivity, including the use of microscopy to detect CPE caused by viral infection (18, 19), plaque assays to quantify infectious virus in the culture supernatants (20), and RT-qPCR to detect increases in viral RNA during culture (21).

To develop a virological endpoint for trials of SARS-CoV-2 therapeutics, it is imperative that all these methodological variables are assessed with the process optimized for maximal sensitivity to enable accurate determination of individuals with infection-competent SARS-CoV-2 in the nasopharynx. In this paper, we describe the optimization of a viral culture assay for detecting infectious virus with assessment of three potential detection methods and three different cell lines.

## MATERIALS AND METHODS

### Variables and experimental design

**Fig 1:**
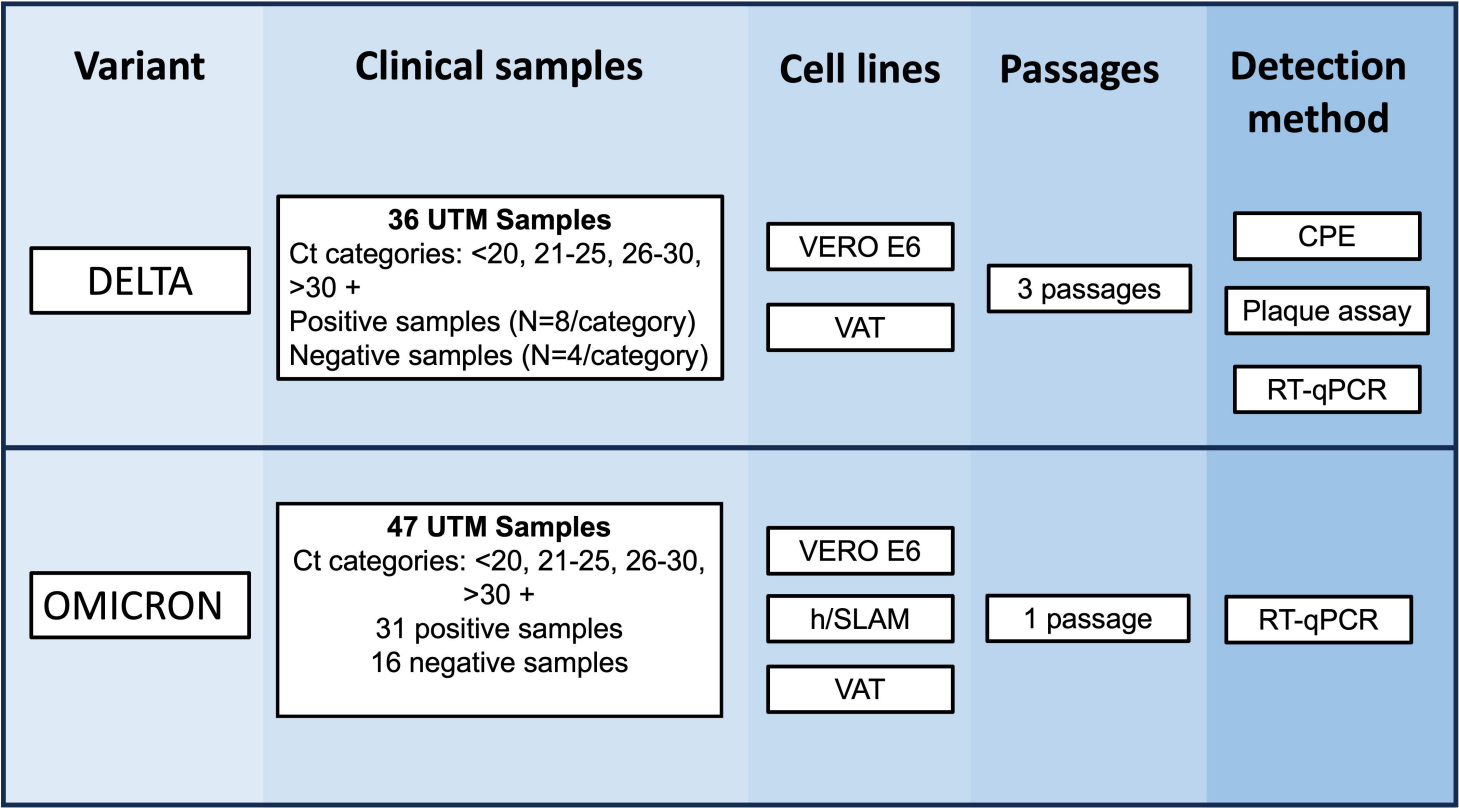
Experimental design for the optimization of SARS-CoV-2 culture from clinical samples. Scheme of the initial experiments performed using different parameters before the optimization of detection methods using clinical samples. Samples during the Delta and Omicron outbreak were cultured in Vero E6, VAT, and/or Vero hSLAM for 3 days. Supernatants were collected to perform reverse-transcriptase PCR for Delta and Omicron, respectively, and CPE imaging and plaque assays were performed for Delta only.

Five variables were identified for optimization (Table 1), and within each variable, a set of parameters was assessed (Fig. 1). Variables were tested using samples from the UK Delta outbreak and, once the methodology was optimized, the procedure was assessed with samples from the Omicron BA.1 outbreak.

**TABLE 1:** Parameters chosen for the optimization of the viral culture assay^*a*^

Variable	Parameters
Sample dilution	1:20, 1:10, and 1:4
Length of viral incubation post-inoculation	24, 48, 72, 96, and 120 hours
Vero cell lines	E6, ACE2/TMPRSS2, and hSLAM
Detection method of viable SARS-CoV-2 virus	CPE, plaque assay, RT-qPCR
Number of viral passages in culture	Passages 1, 2, and 3

^*a*^ CPE, cytopathic effect; RT-qPCR, quantitative reverse transcriptase PCR.

#### Cell culture

Vero C1008 (Vero 76, clone E6, Vero E6) (European Collection of Authenticated Cell Cultures [ECACC] 85020206) (Vero E6 cells) and Vero hSLAM (ECACC 04091501) (hSLAM cells) were obtained from the ECACC. VAT cells were donated from Professor Wendy Barclay (Imperial College London, UK), and their development is detailed in a publication from 2021 (15). Vero E6 cells were maintained in Dulbecco’s modified Eagle medium (DMEM; Gibco, USA) plus 10% fetal bovine serum (FBS; Gibco) and 1% penicillin-streptomycin solution (Gibco) (D10 media). VAT cells were maintained in DMEM plus 10% FBS, 2% 50-mg/mL Geneticin (Gibco), 1% 100× non-essential amino acids (Thermo Fisher, USA), and 0.4% 50-mg/mL Hygromycin B (Invitrogen, USA). hSLAM cells were maintained in Minimum Essential Media GlutaMAX (Gibco) plus 10% FBS and 2% 50-mg/mL Geneticin. All cell lines were maintained at 37°C and 5% CO_2_. Throughout the culture process, these media were required to have their FBS content adjusted to 2% (D2 media) or 4% (D4 media), as detailed below. Cells were passaged every 3–4 days (once cells reached 100% confluency) using trypsin-EDTA solution (Gibco) to dissociate cells from the flask. D10 medium was added to inactivate the trypsin, and cells were re-seeded at a 1:10 ratio, which completed one passage. Cells were maintained for less than 30 passages, so their integrity was not compromised.

The optimal sample volume and number of days growth of virus per passage were determined with the Delta variant of concern (VOC) as stated in the supplemental material (methods a [“Sample dilution”] and b [“SARS-CoV-2 growth curves”]).

#### Plaque assay

The dissociated cell suspension was counted using a Primovert inverted light microscope (ZEISS, Germany) and a disposable C-chip Haemocytometer (NanoEnTek, South Korea). The suspension was diluted with D10 media to a concentration of approximately 250,000 cells/mL, and 500 µL/well was seeded on 24-well plates (Thermo Fisher). The plates were incubated at 37°C + 5% CO_2_ for 18 hours.

Media were discarded, and 190 µL of D2 media was added. Ten microliters of sample was added to the appropriate wells and incubated at 37°C + 5% CO_2_ for 1 hour. An overlay solution (1:1 solution of 2.2% [wt/vol] agarose solution [Sigma-Aldrich, USA] and D4 media) was added to 500 µL/well and incubated at 37°C + 5% CO_2_ for 72 hours. Approximately 1-mL/well of formaldehyde solution (37% [wt/vol]) (Merck, Germany) was added, and the plates were incubated at room temperature for at least 1 hour. The formaldehyde was discarded, and the plates were stained with crystal violet solution (0.25% [wt/vol] in distilled water) for 1 minute, rinsed twice with tap water before air-drying and plaque enumeration.

#### RT-qPCR

RNA was extracted from Delta and Omicron culture supernatants using the QiAamp96 Virus Qiacube HT kit (Qiagen, Germany), and RT-qPCRs were run following the manufacturer’s instruction using TaqPath COVID-19 RT-PCR on a QuantStudio 5 (Thermo Fisher). Fluorescence was recorded in the FAM, VIC, ABY, and JUN channels for the SARS-CoV-2 ORF1ab, N, and S gene targets, plus MS2 RT-qPCR internal control target, respectively. The N gene value was selected as the most stable target to stratify the samples (lower mean Ct and standard deviation at 10 genome equivalent copies/reaction (22). Four and 16 RT-qPCR-confirmed negative samples were also selected as controls in the case of Delta and Omicron clinical samples, respectively.

### SARS-CoV-2 clinical sample cohort

Aliquots of universal transport medium (UTM and UTM-RT; Copan, USA) (nasopharyngeal samples) from a cohort of adult participants with symptoms suggestive of COVID-19 were collected by the “Facilitating Accelerated Clinical Evaluation of Novel Diagnostic Tests for COVID-19 (FALCON C-19), Workstream C (Undifferentiated Community Testing).” Samples were stored at −80°C and thawed for the first time for this study.

#### Initial evaluation

The optimization experiment was carried out using the Delta VOC comparing three detection methods: plaque assay, observable CPE, and comparison of RT-qPCR cycle threshold (23) values before and after viral culture, in all cell lines for three passages. All conditions were tested in triplicate. Methods were evaluated for their sensitivity in detecting culture positivity from RT-qPCR positive samples, with the best performer selected for further testing with Omicron samples.

##### Delta clinical samples

Samples were selected from those collected between 19 July 2021 and 26 October 2021, based on a >99% frequency of the Delta VOC in the UK between these dates (24). Two different sets of Delta-positive clinical samples were used for Vero E6 and VAT cell lines, respectively (*n* = 36, each set) due to the availability of the cell lines at different time points and the requirement of avoiding using freeze-thawed samples. However, samples were selected for the study based on the Ct value obtained when tested at the time of sample collection using the TaqPath COVID-19 RT-PCR kit (Thermo Fisher). Eight samples were selected for each of the following ranges of Ct values: <20, 21–25, 26–30, and >30. Where possible, to ensure a breadth of Ct values within each range, each category was split in half (e.g., <20 was split into 10–15 and 16–20), with four samples taken for each.

##### Impact of viral passage on culture positivity

Cells from each cell line were seeded into 24-well plates. D2 media was added at 190 µL/well, and 10 µL of each UTM sample was added. Plates were incubated at 37°C + 5% CO_2_ for 2 hours, before a further 200 µL/well of D10 media was added. Plates were incubated at 37°C + 5% CO_2_ for 72 hours. The process was repeated twice, with 10 µL/well being transferred to fresh confluent 24-well plates. Each 72-hour incubation was referred to as a viral passage (giving a total of three passages). After each passage, the culture positivity was determined by the methods detailed above.

##### Identification of culture positivity

The three detection methodologies used were microscopy for visual inspection of CPE, plaque assay, and RT-qPCR.

Plaque assays were performed on the 10 µL of supernatant taken from each well at each passage described in “Plaque assay” above. Wells were scored as negative if there were no plaques and positive if there were plaques confirming the presence of an infectious virus. Any plates with detached monolayers after staining were repeated.

The final detection method to assess viral positivity was a comparison of RT-qPCR Ct (23) values from a baseline (the original sample diluted 1:40 using D2 media to replicate the dilution factor inherent for viral culture assessment) and the culture supernatant, using the TaqPath RT-qPCR assay. A decrease in Ct value (meaning an increase of viral genetic material) indicates that the virus has successfully replicated and is therefore present and viable. The reproducibility of the RT-qPCR was determined to further inform the Ct difference selected to be indicative of a positive culture by testing 10 replicates from a unique UTM sample at 1× limit of detection (LOD). The coefficient of variation calculated at 1× LOD was 0.8% (Table S1), and therefore, a positivity cutoff of >1 Ct reduction between baselines and culture supernatants was used.

### Omicron variant

#### Omicron clinical samples

Clinical samples were collected from 14 December 2021 to 28 March 2022 based on >99% frequency of Omicron. Samples were also selected based on previous diagnostic results using the Taqpath RT-qPCR kit, indicating an S gene target failure (25, 26). In addition to the clinical samples, an Omicron virus stock (1.4 × 10^5^ pfu/mL) was also incubated in triplicate as positive control. An aliquot from the same virus stock was heat inactivated at 80°C for 1 hour (27) to generate a negative control, and UTM was used as no virus control.

#### Evaluation of optimized culture methodology

The optimized methodology was determined to be a 3-day incubation of a single passage, a 1:20 sample dilution, and RT-qPCR-based detection. This optimized methodology was validated using 31 positive and 13 negative Omicron samples (Fig. 1). The cell lines Vero E6, VAT, and, in addition, hSLAM cells due to high susceptibility for Omicron infection (28, 29) were used to determine the optimal cell line for this variant. A positive sample (Omicron virus stock), negative sample (inactivated virus stock), and blank (UTM) were used as controls.

### Statistical analysis

Data were collated and analyzed using R (version 4.1.1; R Foundation for Statistical Computing, Vienna). Graphical analysis was undertaken using the ggplot package. The coefficient of variation between replicates and the differences between means on Ct values was obtained using an independent two-sample *t*-test using R (version 4.2.1) (30).

## RESULTS

**Fig 2:**
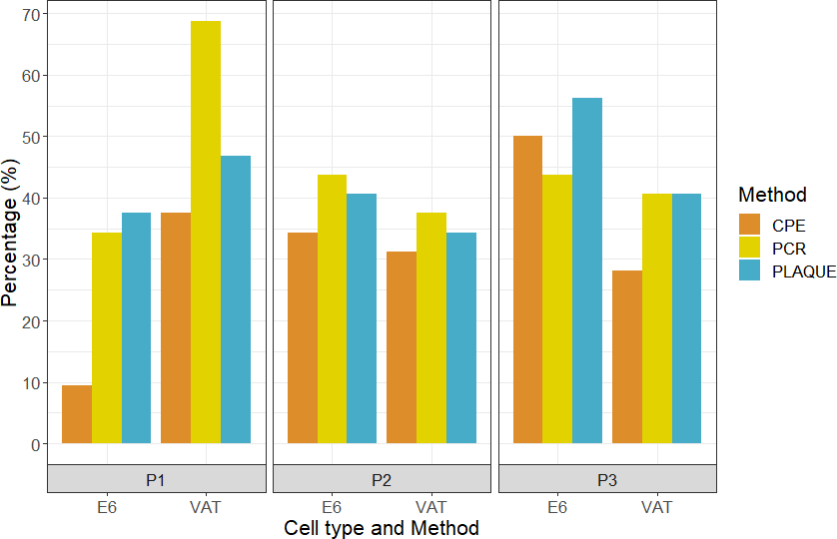
Percentage of positive results by cell type and passage (P1, P2, and P3 refer to passages 1, 2, and 3, respectively) by each detection methodology tested using RT-qPCR-positive samples. PCR denotes RT-qPCR; plaque denotes plaque assay. CPE, cytopathic effect.

Thirty-six nasopharyngeal samples collected during the UK Delta outbreak were used to optimize each methodology. Due to the timings of the experiments and a need to avoid freeze-thaw, different samples were used to assess the Vero E6 and VAT cell lines; however, samples were selected with the same Ct ranges (Fig. 2). There was no significant difference between the mean Ct of the two sample sets (sample set 1 [Vero E6] mean = 25.34, sample set 2 [VAT] mean = 25.48; *P* value = 0.934, CI –3.57 to 3.289). However, the mean Ct of the >30 sample category was higher for sample set 1 than for sample set 2 (35.51 vs 32.46) and significantly different (*P* value = 0.025, CI 0.436–5.667).

### Dilution factor

To reduce the possibility of culture inhibition or contamination while maintaining viable virus detection, the ideal UTM dilution factor was evaluated. There was no significant difference in the number of plaques produced with a sample volume of 10, 20, or 50 µL within each passage of each cell line (Table S2). However, we did observe signs of contamination in some wells of the 20- and 50-µL volumes using both microscopy and visual observation of a change in color of the media. For these reasons, 10 µL of input sample (overall dilution = 1:40) was chosen to reduce the risk of contamination without impacting sensitivity.

#### Optimization results

All SARS-CoV-2-positive clinical samples tested in triplicate by RT-qPCR produced a valid result. Despite dilution of input clinical samples, contamination was still observed in 14 of 576 (2.4%) sample replicates (Table S4A), with 2 and 12 wells contaminated in the E6 and VAT cell line, respectively. In addition, 60 of 576 (13.2%) of the original plaque assays failed due to a lack of viable monolayer and were repeated from a frozen aliquot of the same sample, then added to the final data table (Table S4A). All 60 plaque assay replicate failures were in the E6 cell line.

From virus passage 1, the VAT cells resulted in the highest proportion of positive cultures using each detection method (Fig. 2). The combination that gave the highest number of positive cultures was RT-qPCR with the VAT cells (68.8%). While further passages improved the positivity rate using Vero E6 cells (34.4%, 43.8%, and 43.8% for passages 1, 2, and 3, respectively), the sample positivity in VAT cells decreased across the passages (68.8%, 37.5%, and 40.6% for passages 1, 2, and 3, respectively).

**Fig 3:**
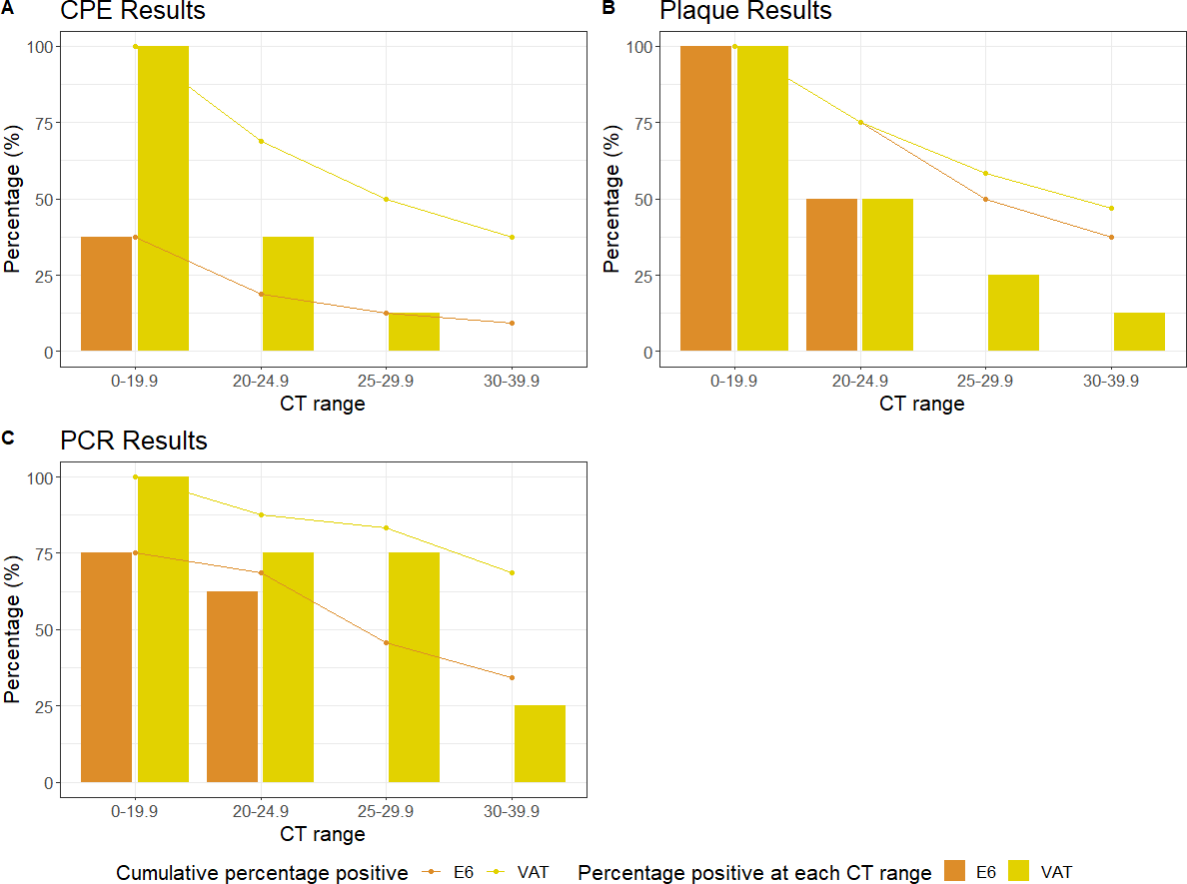
The percentage of positive results according to the different Ct ranges in viral passage 1 using the three detection methods. (A) CPE, (B) plaque assay, and (C) RT-qPCR. CPE, cytopathic effect.

For each cell line at virus passage 1, microscopy was the least sensitive detection method. For samples in the <20 Ct range, detectable CPE yielded a positivity rate of 37.5% and 100% in the Vero E6 and VAT cells, respectively. For samples with Ct values in the 20–40 range, the only positives were found in VAT cells, with a cumulative positivity of 50% for all samples with Ct values of <30 (Fig. 3A). There were no positive samples found at any range for Vero E6 cells except for Ct of <20 (Table S4A).

Plaque assays from culture supernatants identified viable virus in all samples with a Ct of <20 in both cell lines (Fig. 3B). This reduced to 50% in both cell lines for the 20.0–24.9 Ct range. Cumulative positivity rates for each cell line for samples with Ct values of <30 were 50% for E6 cells and 58.3% for VAT cells. RT-qPCR was the most sensitive detection method for passage 1 (Fig. 3C), particularly when using the VAT cells, which had a cumulative positivity rate of 68.75%, 83.3%, 87.5%, and 100.0%, at Ct ranges of and <40, <30, <25, and <20, respectively.

**Fig 4:**
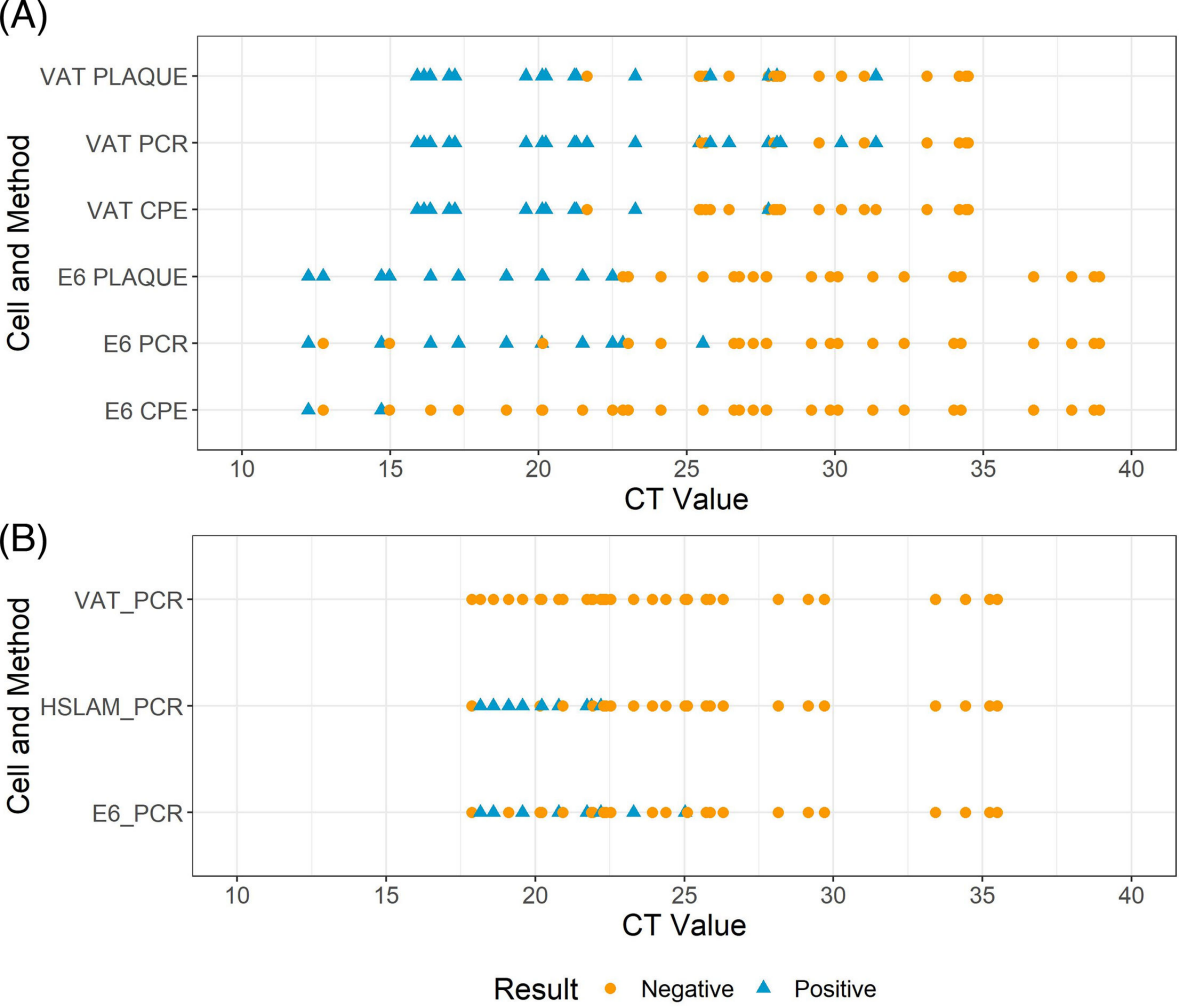
Positive and negative results by original Ct values using the different detection methods with (A) Delta and (B) Omicron samples, respectively. Each dot or triangle represents an individual sample. Most positive results were identified when samples had Ct values of <25 regardless of the variant used. Plaque denotes plaque assay and PCR denotes RT-qPCR. CPE, cytopathic effect.

The VAT cells were the best-performing cell line in terms of sensitivity when using Delta variant-containing samples with lower viral loads (i.e., higher Ct values), with two samples with a Ct value of >30 identified when RT-qPCR was used as a detection method (sample Ct values = 31.79 and 30.20) (Fig. 4A). The sample with the highest Ct detected by Vero E6 from passage 1 was Ct = 25.53. The Vero E6 cells with RT-qPCR combination failed to identify two samples below Ct = 20, with Ct values of 12.74 and 14.97.

Two samples with Ct calues of <20 (6.25%) assessed with VAT cells failed to produce positive results by microscopy (Table S4A). The negative control samples were found to be negative by all assays (Table S5), apart from RT-qPCR from VAT cells (one of four false positive at P3).

Using the final methodology we observed some variation within replicates, particularly with samples with higher Ct values. Of the samples with at least one positive replicate, 67.9% were positive in all replicates, 10.7% in two, and 21.4% in one (Table S6).

### Validation of the final culture protocol using Omicron clinical samples

The optimal protocol from the assay development experiments was determined to be a single 3-day incubation of samples with a 1:20 dilution, and RT-qPCR as the detection method. This was then assessed using 31 positives and 16 negative samples collected during the Omicron outbreak. Three different cell lines: Vero E6, VAT, and hSLAM cells were tested using the same sample set in all cases to assess the optimal cell line for Omicron detection.

### RT-qPCR analysis of viral P1 samples

**Fig 5:**
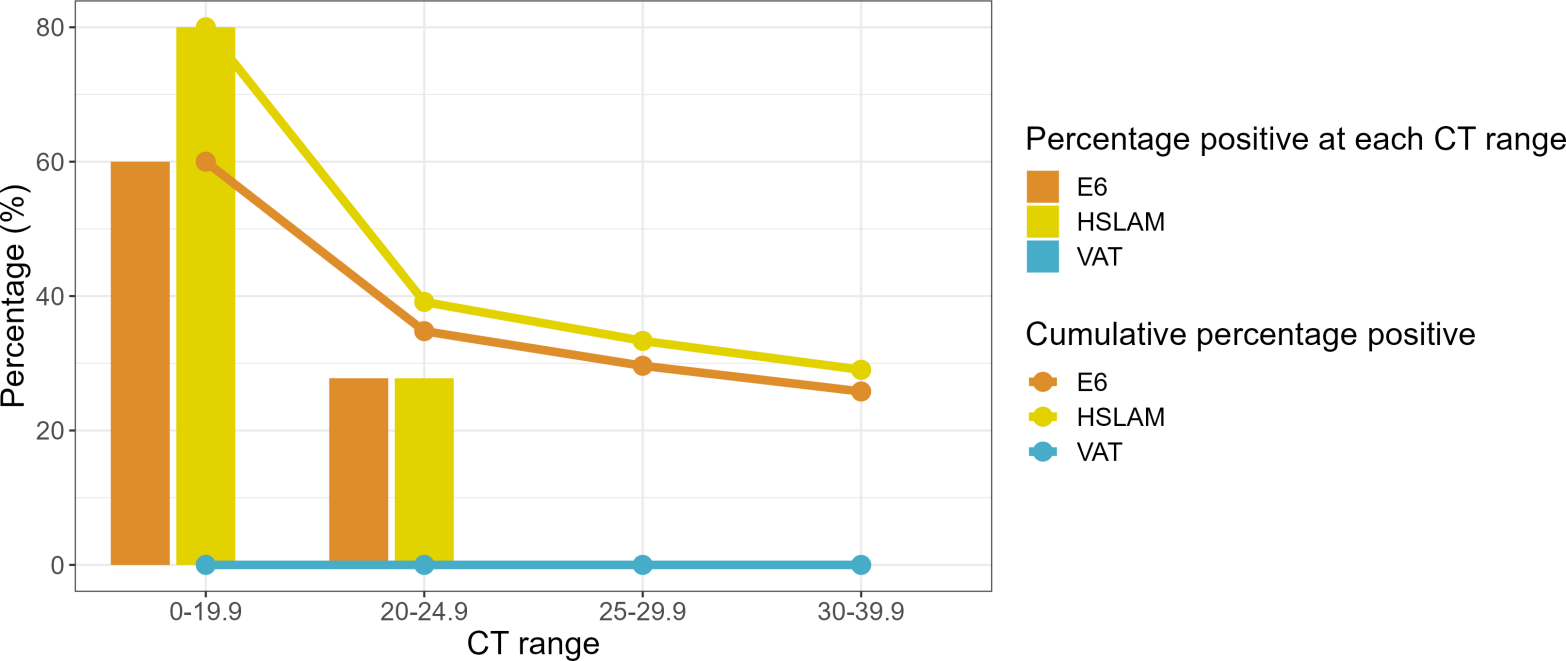
Percentage of Omicron-positive results detected by RT-qPCR according to Ct range. Positive results are presented based on the four different Ct categories for each cell line. Solid colored lines show the cumulative percentage values for each category.

Detection of viable virus was demonstrated to be variable across the three cell lines when assessing passaged clinical samples. Using the optimized protocol, the results showed that both hSLAM and Vero E6 cells resulted in a higher proportion of culture-positive samples than the VAT cells (Fig. 5), which were unable to isolate replicative virus from any of the samples tested. From the 31 Omicron-positive samples used in the study, 7 failed to give a positive result by RT-qPCR in the baseline pre-culture sample after dilution and were therefore treated as negatives. Of the 24 remaining samples, 8 (33.3%), 9 (37.5%), and 0 (0%) were positive when cultured with Vero E6, hSLAM, and VAT cells, respectively.

Overall, culture positivity with the hSLAM and Vero E6 cells was 80% and 60%, respectively, from samples with a Ct value of <20 (Fig. 5). In samples with Ct values of <25, positivity was 35%–40% (Fig. 4B); no sample with a Ct value of >25 was culture positive in any cell line (Table S4B). The RT-qPCR-negative clinical samples were all found to be negative with the optimized culture protocol (13 of 13). The positive control (replicating authenticated virus) showed a mean Ct value of 17.89, 11.67, and 15.60 for Vero E6, hSLAM, and VAT cells when cultured, respectively (Table S7). The negative control and no virus controls were negative regardless of the cell line.

Again, variation was seen within replicates, particularly with samples with higher Ct values. The best-performing cell line, hSLAM, had 69.2% of samples positive in all replicates (Table S6).

## DISCUSSION

The use of cell culture as a virological endpoint in trials of SARS-CoV-2 therapeutics has been limited compared to molecular approaches, due to technical difficulties, lack of standardization, availability of biosafety level 3 laboratories and reduced sensitivity. However, utilizing a culture system to monitor replicating virus could mitigate the potential of molecular methods detecting viral RNA from inactive or lysed viral particles.

Here, we have demonstrated that a combination of VAT and hSLAM cells for viral isolation with RT-qPCR of the culture supernatant at passage 1 for detection is the most sensitive approach for determining the presence of infectious virus of Delta and Omicron VOCs, respectively. Culture positivity was higher overall for samples containing the Delta VOC than Omicron, particularly for those with an RT-qPCR Ct value of >25.

A previous clinical trial of the antiviral drug molnupiravir utilized a cell culture/supernatant RT-qPCR approach, with Vero E6 cells, and identified a replication-competent virus in 43.5% of infected participants at enrollment, a significant difference between control and treatment groups at day 3, plus a dose-response relationship between the drug and viral isolation (5). In the case of clinical samples for Delta VOC, the VAT cells were more sensitive than Vero E6 cells for isolating SARS-CoV-2, particularly from samples with lower viral loads. It is generally regarded that the ACE2 receptor expressed by the VAT cells is critical for the entry of SARS-CoV-2 to human epithelial cells, with the serine protease TMPRSS2 priming the S protein for binding (31). In 2020, the expression of TMPRSS2 by Vero E6 cells has been shown to enhance the isolation of wild-type SARS-CoV-2 from clinical samples (31), which was also apparent in our results with the Delta variant. However, the lack of Omicron viral replication in any clinical sample with the VAT cells was surprising as there was successful viral infection in VAT cells during the growth curve experiments probably due to cell adaptation (albeit with virus passaged through VAT cells previously), and other studies showed Omicron can replicate in VAT cells (32, 33). This experiment took place in parallel with the other cell lines and was further repeated to confirm these findings, with the same results obtained. In contrast, we found that Vero E6 and hSLAM cells were much more effective at isolating virus from Omicron samples. Omicron has low efficiency for TMPRSS-2-mediated cell entry and preferentially infects via cathepsin-mediated endocytosis (29, 32). In addition, it was recently demonstrated that TMPRSS2 activity on both ACE2 and SARS-CoV-2 spike activation is what leads to the significant change in entry requirements from Delta to Omicron lineages (34).

This study focused on viral isolation in Vero cells and their derivatives and did not assess other cell lines reported to be useful culture systems for SARS-CoV-2. While human epithelium-derived cell lines such as Calu-3 and Caco-2 have been used to isolate and propagate SARS-CoV-2, they produce lower viral titers, do not undergo visible CPE or reproducibly allow viral plaques (35), and are less efficient at viral isolation than Vero E6 cells (36, 37). One limitation of our study is that it did not include the Vero hSLAM cell line for isolating the virus from clinical samples containing the Delta VoC, only Omicron.

The use of RT-qPCR to monitor a change in Ct value was more sensitive than microscopy or plaque assay, enabling the detection of replicative virus in the absence of overt CPE. The use of a Ct difference of >1 Ct for culture positivity was selected based on the reproducibility of the assay, and the prediction that RNA from non-replicative virus would decay during incubation; however, an increased Ct difference could be selected to increase specificity while potentially reducing sensitivity. Due to failed controls on plates (e.g., the virus free control having a disrupted monolayer, making result interpretation impossible), the plaque assay approach produced a substantial proportion of unsuccessful replicates, resulting in the need for repeats. While RT-qPCR is the most sensitive and rapid analytical technique used, the data generated require longitudinal analysis to demonstrate the presence of a replicating virus.

The methodologies reported utilized 24-well plates to provide a reasonable throughput while enabling sufficient inoculum volume to maintain sensitivity. These assays could potentially be carried out in 48- or 96-well plates to maximize throughput or scaled up to 6-well plates or flasks to maximize input volume and potential sensitivity. While we added antibiotics and antimycotics to cell culture media to reduce contamination, filtering the inoculum could lessen the need for inoculum dilution, thereby benefitting sensitivity. Other culture-based methods such as TCID_50_ assays can be done at a higher throughput than plaque assays; however, these were not evaluated during our method development.

The specificity of the assay was found to be high, with the only false positives found in passage 3 by RT-qPCR for Delta and no false positives for Omicron. This could have been due to contamination of the cell culture or RT-qPCR assays. The study only included four SARS-CoV-2-negative swab samples for Delta analysis, and further testing is required to assess the test specificity more confidently. In the case of analysis against Omicron clinical samples, a greater number of negative samples were included (*n* = 16).

Despite lower sensitivity than RT-qPCR at detecting the presence of SARS-CoV-2 in clinical samples, the greater specificity of cell culture, which only detects viable virus, improves the ability to evaluate efficacy of an antiviral by revealing a larger difference between the treatment and control arms. Infection and viral load kinetics differ between SARS-CoV-2 variants, and this work reinforces the need to verify cell line suitability for circulating variants before the selection of cell line for culture-based diagnostics. Even though more effort and caution are required as variations alter, the improved data produced may benefit research projects such as clinical trials.

Here we have optimized a cell culture-based assay for determining the presence of infectious SARS-CoV-2 Delta and Omicron variants using clinical nasopharyngeal swabs, determining the optimal sample dilution, culture time, cell line, passage, and detection method. We have also identified an ongoing need to periodically re-assess optimal cell lines throughout the pandemic as the virus evolves and receptor usage and tropism change over time. This methodology may have application as a secondary virological endpoint in clinical trials of therapeutics for SARS-CoV-2, in addition to numerous research processes.

## SUPPLEMENTAL MATERIAL

Supplemental material msphere.00304-24-s0001.pdf Supplemental text, figures, and tables.

ASM does not own the copyrights to Supplemental Material that may be linked to, or accessed through, an article. The authors have granted ASM a non-exclusive, world-wide license to publish the Supplemental Material files. Please contact the corresponding author directly for reuse.
